# Evaluating indices of body condition in two cricket species

**DOI:** 10.1002/ece3.1257

**Published:** 2014-11-11

**Authors:** Clint D Kelly, Brittany R Tawes, Amy M Worthington

**Affiliations:** 1 Département des Sciences Biologiques, Université du Québec à MontréalCP-8888 Succursale Centre-ville, Montréal, Quebec, Canada, H3C 3P8; 2 Department of Ecology, Evolution & Organismal Biology, Iowa State UniveristyAmes, Iowa, 50011

**Keywords:** Body condition, body condition index, energy stores, fat content, residual mass, scaled mass index

## Abstract

Body mass components (dry mass, lean dry mass, water mass, fat mass) in each sex correlate strongly with body mass and pronotum length in *Gryllus texensis* and *Acheta domesticus*. Ordinary least squares (OLS) regression underestimates the scaling relationship between body mass and structural size (i.e., pronotum length) in both cricket species compared with standard major axis (SMA) regression. Standardized mass components correlate more strongly with scaled mass index (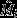) than with residual body mass (*R*_i_). *R*_i_ represents the residuals from an OLS regression of log body mass against log pronotum length. Neither condition index predicts energy stores (i.e., fat content) in *G. texensis*. *R*_i_ is not correlated with energy stores in *A. domesticus* whereas 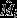 is negatively correlated. A comparison of condition index methods using published data showed that neither sex nor diet quality affected body condition at adulthood in *G. texensis* when using the scaled mass index. However, the residual index suggested that sex had a significant effect on body condition. Further, analysis of covariance (ANCOVA) suggested that diet quality significantly affects body mass while statistically controlling for body size (i.e., body condition). We conclude that the statistical assumptions of condition index methods must be met prior to use and urge caution when using methods that are based on least squares in the *y* -plane (i.e., residual index ANCOVA).

## Introduction

Body condition refers to an animal's energetic state and is generally considered to be an indicator of an animal's health, quality, and vigor (Schulte-Hostedde et al. 2001; Tomkins et al. 2004; Schulte-Hostedde et al. 2005; Peig and Green 2009; Cox et al. 2014; see also Rowe and Houle 1996). Evolutionary ecologists are particularly interested in body condition because many sexually selected, and life history traits are condition dependent (Andersson 1994; Lochmiller and Deerenberg 2000; Tomkins et al. 2004). Accurately quantifying body condition often involves measuring the relative amount of fat stored by an individual (e.g., Gray and Eckhardt 2001; Williams and Robertson 2008; Kelly 2011) because it is the primary fuel for fitness-related processes such as immunity (Demas et al. 2011) and the performance of sexually selected traits and behaviors (Andersson 1994; Bradbury and Vehrencamp 2011).

Directly measuring fat content is often unappealing because it requires the destruction of the study animal (Stevenson and Woods 2006; Williams and Robertson 2008; Schamber et al. 2009). Consequently, evolutionary ecologists have employed a number of non-destructive methods to serve as indices of body condition (Sears 1988; Redfern et al. 2000; Sutton et al. 2000; Stevenson and Woods 2006; Peig and Green 2009), with the residual index being the most common method used. Residual index (*R*_i_) is calculated for each individual as the residual from an ordinary least squares (OLS) regression of body mass on a measurement of length (e.g., Jakob et al. 1996; Schulte-Hostedde et al. 2001, 2005; Ardia 2005). This method, however, has several well-documented caveats (Kotiaho 1999; Garcia-Berthou 2001; Green 2001; Peig and Green 2009, 2010), including the assumption that the unexplained variance in body mass represents variance in the appropriate pool of resources (e.g., fat content).

Peig and Green (2009, 2010) showed that conventional methods can be inherently biased with regard to animal size and tend to change condition scores in larger animals owing to violations of statistical assumptions and failure to account for growth and scaling relationships. To overcome this problem, they developed the scaled mass index (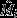; Peig and Green 2009), which accounts for the covariation between body size and body mass components in the calculation of a condition score by standardizing body mass at a fixed value of a linear body measurement based on the scaling relationship between mass and length.

Another popular approach is to conduct an analysis of covariance (ANCOVA), which combines features of linear regression and analysis of variance to directly estimate the treatment effect on body mass while statistically controlling for a concomitant variable of influence, which is generally a measurement of body length (Garcia-Berthou 2001; Serrano et al. 2008). The ANCOVA is not strictly a condition index, but rather an inferential statistical test where individual condition scores are absent, thus making validation via correlations with body components, such as fat reserves, impossible. Several studies have made use of this technique (e.g., Velando and Alonso-Alvarez 2003; Lendvai et al. 2007; Sztatecsny et al. 2013).

No matter which condition index or method is used, its ability to predict fat content (or the mass component of interest) must be empirically verified against measured values of fat (Kotiaho 1999; Rolff and Joop 2002; Schamber et al. 2009). Several studies have shown that the residuals from an OLS regression of body mass on body size correlate with the absolute size of an individual's energy stores (e.g., Cavallini 1996; Schulte-Hostedde et al. 2001; Cattet et al. 2002; Ardia 2005; Schamber et al. 2009). Peig and Green (2009) argue, however, that such correlations are not surprising because OLS residuals are biased toward larger individuals and larger individuals tend to also have larger absolute fat stores. Other investigators have shown that residual body mass correlates with relative fat content (Weatherhead and Brown 1996; Wirsing et al. 2002), but this approach has been criticized on statistical grounds because it assumes an isometric relationship between different body components (Kotiaho 1999; Peig and Green 2009). In their re-analysis of the data published in three of the previously cited vertebrate studies (i.e., Weatherhead and Brown 1996; Schulte-Hostedde et al. 2001; Ardia 2005), Peig and Green (2010) showed that 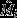 performs better than *R*_i_ as a predictor of variation in fat reserves as well as other body components. We do not know, however, how 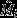 performs relative to *R*_i_ in any insect. We address this issue here in two species of gryllid cricket (Orthoptera).

Gryllid field crickets are popular model organisms in studies of evolutionary ecology and body condition is a common factor of interest in these investigations. For example, empirical tests have examined how body (or nutritional) condition mediates the expression of male sexual signals (Gray and Eckhardt 2001; Scheuber et al. 2003, 2004; Hunt et al. 2004; Judge et al. 2008; Tolle and Wagner 2011; Whattam and Bertram 2011; Bertram and Rook 2012), male dominance and fighting ability (Bertram and Rook 2012), the quality of male ejaculates (Andrade and Mason 2000; Simmons 2012), and immunocompetence and disease resistance (Jacot et al. 2004; Kelly and Tawes 2013). To date, a handful of workers have quantified body condition in gryllid species directly by measuring fat content (Gray and Eckhardt 2001; Worthington et al. 2013). Most workers, however, have assessed body condition by measuring body mass independent of body size (Hunt et al. 2004, 2005; Judge et al. 2008) or using residual body mass (Wagner and Hoback 1999; Andrade and Mason 2000; Whattam and Bertram 2011; Harrison et al. 2013), with only a few adopting the newly developed scaled mass index (e.g., Kelly and Tawes 2013; Stahlschmidt et al. 2013; Kelly et al. 2014).

Our overall objectives in this paper are to identify a condition index that is, a reliable indicator of energetic condition (i.e., fat content) in two cricket species, *Gryllus texensis* and *Acheta domesticus*, that are commonly used in studies of evolutionary ecology, and to critically compare the performance of different indices whose use is advocated in the literature: *R*_i_ (Jakob et al. 1996; Marshall et al. 1999), 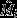 (Peig and Green 2009, 2010), and ANCOVA (Garcia-Berthou 2001). We achieve the latter goal using data previously presented in Kelly and Tawes (2013) on the effect of sex and nutritional conditions during development on body condition at adulthood in *G. texensis*. An additional goal of this study is to add invertebrate taxa to the vertebrate-dominated list of animals in which condition indices have been verified. It is essential to examine these issues in invertebrates for the simple fact that their unique biology could affect scaling relationships between measures of body size and body mass components, particularly fat content.

## Methods and Materials

Large populations were established in the laboratory for *G. texensis* and *A. domesticus*. These populations comprised animals that were raised from hatching under identical and optimal environmental conditions and thus gave true estimates of the mass–length relationship (hereafter referred to as reference populations; Peig and Green 2010). These reference populations were used to determine correlations among mass components (absolute and relative mass) and body size measures, determine mass–length scaling relationships (i.e., *b*_SMA_, see below), and to examine how mass components correlate with condition scores from residual analysis and the scaled mass index.

The reference data set for *G. texensis* (*n*  = 86 males; *n*  = 103 females) comprised lab-reared descendants of crickets collected in Austin, TX (U.S.A.). These crickets were raised communally for their first 3 weeks in large bins (64 L) and then housed individually in 250-mL containers (10 cm diameter × 4.5 cm depth) until eclosion to adulthood. The reference data set for *A. domesticus* (*n*  = 59 males; *n*  = 61 females) comprised the crickets used in Worthington et al. (2013). Juvenile crickets (4–5 weeks of age) were acquired from a commercial dealer (Fluker's Cricket Farms, Port Allen, LA), and the sexes were separated prior to their imaginal molt, with females housed in large communal bins (44 × 33 × 40 cm) and males housed individually in 250-mL containers (10 cm diameter × 4.5 cm depth). For both species, containers were cleaned weekly and all individuals were provided with cotton-plugged water vials and fed dry cat food (Special Kitty: 34% protein, 13% fat) ad libitum. Crickets were reared and maintained at 27 ± 1°C on a 12 h:12 h light:dark cycle and were checked daily for eclosion to adulthood.

Adult crickets were euthanized by freezing at −20°C either at eclosion (*G. texensis*) or 12–14 days post-eclosion (*A. domesticus*). Body mass (g) and pronotum length (mm) were recorded immediately after death in both species with the lengths of the left and right tibia and femura also being recorded in *G. texensis*. Pronotum length was defined as the distance between the anterior and posterior edges of the pronotum. Pronota, tibia and femura were measured to the nearest 0.01 mm under a stereomicroscope using Leica LAS image analysis software (Leica Microsystems Inc., Buffalo Grove, IL).

Crickets were dried at 60°C for 24 h and weighed to the nearest 0.01 mg using an electronic balance (Denver Instruments TP-64). Water mass was measured as the difference between fresh mass and dry mass. Body fat was then extracted using petroleum ether (Fisher Scientific, Hanover Park, Illinois, USA) reflux in a Soxhlet apparatus for 12 h. Individuals were again dried at 60°C for 24 h and then reweighed to obtain their lean dry mass. Body fat content (mg) was obtained by subtracting lean dry mass from dry mass.

For both species, we used Pearson product-moment correlation (*r*) to assess the strength of the relationship of absolute and relative mass components with body size measures for males and females separately as well as both sexes pooled. Relative (%) mass components were calculated as mass component divided by size measure multiplied by 100.

### Body condition validation

Body condition at eclosion was calculated for each individual using Peig and Green's (2009) scaled mass index. This index standardizes body mass to a specific fixed value of a linear body measurement based on the scaling relationship between mass and length using the equation:


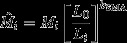
(1)

where *M*_i_ and *L*_i_ are the body mass and linear body measurement of individual *i*, respectively, *b*_SMA_ is the scaling exponent estimated by the standardized major axis (SMA) regression of ln *M* on ln *L*; *L*_0_ is an arbitrary value of *L* (e.g., the arithmetic mean value for the study population); and 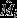 is the predicted body mass for individual *i* when the linear body measure is standardized to *L*_0_.

In our *G. texensis* reference population, log body mass is positively correlated with log femur length (*r * = * * 0.6467, *P * < * * 0.0001, *n*  = 189) and log tibia length (*r * = * * 0.5635, *P * < * * 0.0001, *n*  = 189), but is most strongly correlated with log pronotum length (*r * = * * 0.8326, *P * < * * 0.0001, *n*  = 189). In our *A. domesticus* reference population, log body mass is also strongly positively correlated with log pronotum length (*r * = * * 0.7332, *P * < * * 0.0001, *n*  = 121). Therefore, pronotum length is an excellent linear indicator of body size in both cricket species and was used as *L* in our calculations of 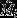 (*G. texensis*: *L*_0_ = 3.349 mm; *A. domesticus*: *L*_0_ = 2.908 mm).

For each of our reference populations, we first used model II regression to calculate the allometric slope (*b*_SMA_) of the best-fit line from a standardized major axis regression of fresh body mass on pronotum length (both variables log-transformed). The scaling mass index is superior to other methods of determining body condition from mass and length estimates because its use of model II linear regression (i.e., standardized major axis regression, henceforth SMA). SMA is superior to other regression techniques when, for example, both variables have some underlying error rate associated with their measurement and are measured on different scales (Warton et al. 2006; Peig and Green 2009). The model II slopes did not differ between the sexes in either species (see Results and Discussion), and so a common slope (*G. texensis*: *b*_SMA_ = 2.642; *A. domesticus*: *b*_SMA_ = 2.549) was calculated for each species. For each species, we calculated each individual's 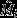 by substituting the appropriate slope and mean pronotum length (*G. texensis*: *L*_0_ = 3.349 mm; *A. domesticus*: *L*_0_ = 2.908 mm) into Eq. (1) along with each individual's fresh body mass (*M*_i_) and pronotum length (*L*_i_).

We used the same method to standardize the other body components (i.e., fat content, dry mass, lean dry mass, and water content) for a fixed size (*M*_i_ in Eq. 1 was taken to be the mass of the component). Peig and Green (2009) recommended such standardization because body components (e.g., fat, protein, water, etc.) are generally correlated with body size. We note that the same *L*_0_ value (i.e., pronotum length for *G. texensis*: *L*_0_ = 3.349 mm; *A. domesticus*: *L*_0_ = 2.908 mm) was used for both the scaled component mass and the scaled body mass index.

Residual index (*R*_i_), for each cricket in the reference population, was calculated by entering log body mass as the dependent variable into an ordinary least squares (OLS) regression model with log pronotum length as the independent variable. The standardized residual was then extracted for each cricket. Prior to computing the common slope, we also tested whether the slopes differed between the sexes and diet treatments. Separate analyses of covariance were used to test whether the elevations (i.e., adjusted means) of the slopes differed between the sexes and diets.

We correlated scaled mass components with 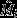 and *R*_i_ for females, males, and both sexes pooled using Pearson product-moment correlation (*r*).

### Comparing methods

We used data presented in Kelly and Tawes (2013) to compare the performance of the scaled mass index with that of two other commonly used approaches (residual index and ANCOVA). Kelly and Tawes (2013) examined the interaction between nutritional quality (poor vs. good diet) during development and sex on various fitness-related traits, including body condition, at adulthood. This data set comprised information on the body size (pronotum length, mm) and body mass (g) at eclosion for *n*  = 82 females and *n*  = 92 males (see Kelly and Tawes 2013 for details).

Kelly and Tawes (2013) calculated condition scores using 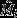, but the allometric scaling exponent they used was calculated from that data set (*b*_SMA_ = 2.319). In this study, we calculated 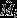 for Kelly and Tawes’ (2013) crickets using the *b*_SMA_ from the *G. texensis* reference population (*b*_SMA_ = 2.642; see above), while the mean pronotum length was the same in both cases (i.e., *L*_0_** = * * 3.073 mm). As discussed by Peig and Green (2010), using the *b*_SMA_ from the experimental population (e.g., Kelly and Tawes 2013) might not be ideal because the development of the test animals was manipulated via diet restriction and thus they might not exhibit the “true” scaling relationship. We substituted these values along with individual body mass (*M*_i_) and pronotum length (*L*_i_) into Eq. (1) to calculate 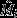 for each cricket. Prior to calculating the common standard major axis regression slope (*b*_SMA_) for use in Eq. (1), however, we first tested the assumption that the slopes did not differ between the sexes and diet treatments by adding either sex or diet to the model and inspecting the interaction term (a significant interaction suggests that the slopes are heterogeneous). Similarly, we tested whether the elevations of standard major axis slopes differed within each factor by inspecting their 95% confidence intervals; the hypothesis of different elevations is rejected by overlapping confidence intervals. All variables were log-transformed prior to analysis. We tested the effect of sex and diet on 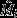 by entering both of these fixed factors as independent variables into an ANOVA.

We calculated *R*_i_ by entering the dependent variable log body mass into an OLS regression model with log pronotum length as the independent variable. The standardized residual was then extracted for each cricket. Prior to computing the common slope (*b*_OLS_) we tested whether the slopes differed between the sexes and diet treatments. Separate analyses of covariance were used to test whether the elevations (i.e., adjusted means) of the slopes differed between the sexes and diets. *R*_i_ was then entered into an ANOVA as the dependent variable with sex and diet treatment entered as fixed-factor treatment variables.

We assessed the performance of ANCOVA by entering log-transformed body mass into a general linear model as the dependent variable with sex and diet treatment as fixed-factor independent variables and log pronotum length as a covariate. This procedure first required testing the homogeneity of slopes assumption; if the interactions between sex and log pronotum length, and diet and log pronotum length were statistically non-significant they were removed and the ANCOVA performed.

For our analyses of 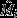 and *R*_i_, we tested for homoscedascity among the condition scores within each treatment using Levene's test. All statistical analyses were performed in R 3.0.2 (R Development Core Team 2014) using the packages *lmodel2* (Legendre 2013), *smatr* (Warton et al. 2011), and *ggplot2* (Wickham 2009). All statistical tests were conducted at the *α*  = 0.05 level.

## Results and Discussion

### Correlations between components of body mass and body size

Validating a condition index by correlating either absolute or relative (i.e., percentages) size of body mass components with a measure of structural size can be misleading in the absence of isometry either because a lack of variation in body size is assumed (absolute values) or because the size of different components can scale differently with increasing total body size (relative values; Kotiaho 1999). Therefore, the scaling relationship between body mass components and body size must be taken into account when validating a condition index (Peig and Green 2009).

This problem is highlighted by our correlations between body mass components and body mass (*M*) and linear size measurements (*L*) in our reference populations (Table1). Our pooled data showed that absolute values of most components positively correlated with each of the *M* and *L* variables in both species; however, fat mass was negatively correlated with body size measures in *A. domesticus*. The strongest correlations in *G. texensis* were between mass components and body mass and pronotum length; body mass and pronotum length also correlated strongly with mass components in *A. domesticus*. Because both *M* and *L* variables are potentially indicative of body size, this suggests that mass components were generally dependent on total body size.

**Table 1:** Correlations between body mass composition (absolute [g] or relative [%] amount) with body mass (g) and linear body measurements (pronotum length, tibia length, femur length, mm) for reference populations of *Gryllus texensis* and *Acheta domesticus* crickets (significant relationships at *P * < * * 0.05 are in bold). Neither tibia nor femur length were measured for *A. domesticus* (see Worthington et al. 2013)

	Sex	Body mass		Pronotum length		Tibia length		Femur length	
		*r*	*P*	*r*	*P*	*r*	*P*	*r*	*P*
*G. texensis*									
Dry mass	Pooled	**0.955**	<0.001	**0.840**	<0.001	**0.634**	<0.001	**0.714**	<0.001
	Female	**0.957**	<0.001	**0.862**	<0.001	**0.659**	<0.001	**0.755**	<0.001
	Male	**0.917**	<0.001	**0.782**	<0.001	**0.742**	<0.001	**0.776**	<0.001
Fat mass	Pooled	**0.160**	0.0270	**0.326**	<0.001	**0.273**	<0.001	**0.338**	<0.001
	Female	**0.299**	0.002	**0.440**	<0.001	**0.247**	0.012	**0.338**	<0.001
	Male	**0.652**	<0.001	**0.560**	<0.001	**0.464**	<0.001	**0.551**	<0.001
Lean dry mass	Pooled	**0.949**	<0.001	**0.745**	<0.001	**0.549**	<0.001	**0.604**	<0.001
	Female	**0.970**	<0.001	**0.788**	<0.001	**0.648**	<0.001	**0.715**	<0.001
	Male	**0.948**	<0.001	**0.804**	<0.001	**0.826**	<0.001	**0.801**	<0.001
Water mass	Pooled	**0.979**	<0.001	**0.782**	<0.001	**0.603**	<0.001	**0.676**	<0.001
	Female	**0.981**	<0.001	**0.769**	<0.001	**0.613**	<0.001	**0.712**	<0.001
	Male	**0.947**	<0.001	**0.764**	<0.001	**0.759**	<0.001	**0.743**	<0.001
% Dry mass	Pooled	0.102	0.163	**0.219**	0.002	**0.157**	0.031	**0.169**	0.020
	Female	−0.300	0.761	**0.210**	0.033	0.115	0.249	0.095	0.341
	Male	**0.212**	0.049	**0.230**	0.033	**0.221**	0.040	**0.269**	0.012
% Fat mass	Pooled	−**0.208**	0.004	0.008	0.909	0.045	0.537	0.075	0.303
	Female	−0.039	0.696	0.172	0.083	0.037	0.707	0.084	0.395
	Male	**0.341**	0.001	**0.303**	0.004	**0.225**	0.037	**0.316**	0.003
% Lean dry mass	Pooled	**0.389**	<0.001	**0.208**	0.004	0.095	0.191	0.066	0.364
	Female	0.033	0.738	−0.036	0.729	0.092	0.354	−0.030	0.760
	Male	−**0.276**	0.010	−0.159	0.144	−0.012	0.910	−0.105	0.334
% Water mass	Pooled	−0.102	0.163	−**0.219**	0.002	−**0.157**	0.031	−**0.169**	0.019
	Female	−0.212	0.050	−**0.210**	0.033	−0.115	0.250	−0.095	0.341
	Male	0.030	0.761	−**0.230**	0.033	−**0.221**	0.040	−**0.269**	0.012
*A. domesticus*									
Dry mass	Pooled	**0.534**	<0.001	**0.429**	<0.001				
	Female	**0.321**	<0.001	**0.215**	0.011				
	Male	**0.457**	<0.001	**0.378**	0.003				
Fat mass	Pooled	−**0.438**	<0.001	−**0.333**	<0.001				
	Female	0.156	0.230	0.148	0.256				
	Male	0.167	0.205	0.018	0.894				
Lean dry mass	Pooled	**0.731**	<0.0001	**0.559**	<0.001				
	Female	**0.941**	<0.001	**0.430**	<0.001				
	Male	**0.392**	0.002	**0.487**	<0.001				
Water mass	Pooled	**0.915**	<0.001	**0.640**	<0.0001				
	Female	**0.983**	<0.001	**0.528**	<0.0001				
	Male	**0.450**	<0.001	0.172	0.193				
% Dry mass	Pooled	−0.044	0.539	0.052	0.462				
	Female	−**0.224**	0.008	−0.082	0.334				
	Male	−0.131	0.318	0.049	0.709				
% Fat mass	Pooled	−**0.577**	<0.001	−**0.458**	<0.001				
	Female	−0.123	0.346	−0.019	0.884				
	Male	−0.071	0.594	−0.143	0.280				
% Lean dry mass	Pooled	−**0.244**	0.007	−0.104	0.257				
	Female	**0.941**	<0.001	0.123	0.344				
	Male	−0.088	0.507	0.206	0.117				
% Water mass	Pooled	**0.553**	<0.001	**0.381**	<0.001				
	Female	−**0.460**	<0.001	−0.142	0.277				
	Male	0.129	0.328	−0.086	0.517				

Examining the sexes separately in *G. texensis* shows that correlations based on absolute values are consistent in direction across the sexes, but differ in strength (Table1). For example, fat mass positively correlates with *M* and *L* in both sexes, but is more strongly correlated in males than in females. In *A. domesticus*, on the other hand, neither *M* nor *L* correlates with fat mass in either sex (Table1). In one of the few studies that has examined sex differences in the relationship between fat content and body size, Rolff and Joop (2002) found that in the damselfly *Coenagrion puella* body mass (fresh weight) positively correlated with fat load in females only. In a study of newly emerged female Douglas fir beetles *Dendroctonus pseudotsugae* (Hopkins) Williams and Robertson (2008) found that body mass was significantly correlated with fat load.

Correlations involving relative component size, on the other hand, were inconsistent across species and between the sexes within species, in both direction and magnitude (Table1). This suggests that scaling relationships between different body mass components and different measures of body size are species and sex specific. Our data reinforce the notion that a reliable condition index for crickets must account for the scaling relationship between different body size measures and mass components, something that *R*_i_ cannot achieve.

### Validating condition indices as predictors of condition

The sexes did not differ in their OLS slopes in either of our reference populations (*G. texensis*: pronotum × sex interaction, *t * = * * 0.067, df = 185, *P * = * * 0.947; *A. domesticus*: pronotum × sex interaction, *t * = * * 0.072, df = 116, *P * = * * 0.943), which justifies examining the scaling relationship between body mass and body size using a common slope (i.e., sex-pooled data; *G. texensis*: *b*_OLS_ = 2.20; *A. domesticus*: *b*_OLS_ = 1.857). Similarly, we found no sex difference in SMA slopes in *G. texensis* (males vs. females: *b*_SMA_ = 2.39 [2.11–2.69] vs. *b*_SMA_ = 2.34 [2.10–2.61], Log likelihood ratio = 0.051, *N * = * * 189, *P * = * * 0.82) or in *A. domesticus* (males vs. females: *b*_SMA_ = 1.64 [1.35–2.00] vs. *b*_SMA_ = 2.10 [1.69–2.61], Log likelihood ratio = 2.272, *N * = * * 120, *P * = * * 0.099) therefore justifying the use of a common slope (*G. texensis*: *b*_SMA_ = 2.642; *A. domesticus*: *b*_SMA_ = 2.549). These latter two slopes were substituted for *b*_SMA_ in Eq. (1).

**Figure 1:**
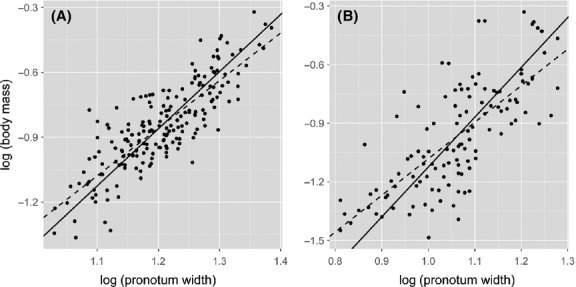
Computed slopes (95% confidence interval) from ordinary least squares (OLS; dashed line) and standard major axis (SMA; solid line) regression of body mass (g) against pronotum length (mm; both variables log-transformed) in (A) *Gryllus texensis* (b_SMA_ = 2.642 [2.439–2.862], b_OLS_ = 2.20 [1.988–2.411]) and (B) *Acheta domesticus* (*b*_SMA_ = 2.542 [2.245–2.878], *b*_OLS_ = 1.857 [1.540–2.173]). OLS regression significantly underestimated the relationship between body mass and body size in both species (see text).

Comparison of the SMA and OLS slopes showed that OLS regression significantly underestimated the slope between log body mass and log pronotum length in *G. texensis* (*b*_SMA_ = 2.642, *b*_OLS_ = 2.20; difference between slopes: *t * = * * 2.92, df = 185, *P * = * * 0.0039) and in *A. domesticus* (*b*_SMA_ = 2.549, *b*_OLS_ = 1.857; *t * = * * 3.03, df = 116, *P * = * * 0.003; Fig.1) suggesting that SMA better describes the scaling relationship between body mass and body size than OLS regression. Lower slope estimates (i.e., those derived from our OLS regressions) will tend to overestimate the condition of larger individuals (i.e., larger positive residuals for larger individuals; Fig.1). The SMA slopes for our reference populations were similar to those found in other studies on crickets (Kelly and Tawes 2013; Kelly et al. 2014). Although these slopes were lower than the value of 3.0 that is, predicted under simple geometric scaling (Green 2001) they were closer to 3.0 than the OLS estimates, which suggests that SMA better describes the ‘true” scaling relationship between *M*_i_ and *L*_i_. Deviation from 3.0 is common across animals and arises because growth is rarely isometric (Peig and Green 2009).

Our pooled data showed that, the scaled mass index, 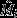, was more strongly correlated with each of the scaled mass components than was residual body mass, *R*_i_. However, neither condition index predicted fat content in *G. texensis*, while only 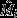, was significantly (but negatively) related to fat content in *A. domesticus* (Table2). Our data therefore suggest that the scaling relationship between different body mass components is properly accounted for by Eq. 1, and that 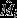 explains more of the variance in individual body mass components than OLS residuals. Similarly, Peig and Green (2009) found that 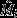 was consistently better correlated with other standardized components (lean dry mass, water, protein, and ash) than OLS residuals in seven vertebrate species. Schamber et al. (2009) also found that the ability to predict fat content in waterfowl varied with the type of condition index used and species studied.

**Table 2:** Correlations between the scaled mass index (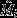) or residual index (*R*_i_, from a regression of ln body mass on ln pronotum length) and each of four different body mass components (g) for the sexes separately and pooled in *Gryllus texensis* and *Acheta domesticus*. Both condition indices were correlated with component mass scaled according to Eq. 1. Significant differences at *P * < * * 0.05 are in bold. Dry mass = no water; lean dry mass = no water or fat

Scaled mass component	Sex	Scaled mass index ( 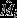 )		Ordinary least squares residuals (*R*_i_)	
		*r*	*P*	*r*	*P*
*G. texensis*					
Dry mass	Pooled	**0.857**	<0.001	**0.800**	<0.001
	Female	**0.887**	<0.001	**0.796**	<0.001
	Male	**0.799**	<0.001	**0.766**	<0.001
Fat mass	Pooled	0.056	0.443	−0.116	0.112
	Female	0.069	0.485	−0.099	0.316
	Male	**0.574**	<0.001	**0.435**	<0.001
Lean dry mass	Pooled	**0.899**	<0.001	**0.824**	<0.001
	Female	**0.931**	<0.001	**0.803**	<0.001
	Male	**0.848**	<0.001	**0.707**	<0.001
Water mass	Pooled	**0.948**	<0.001	**0.885**	<0.001
	Female	**0.972**	<0.001	**0.922**	<0.001
	Male	**0.896**	<0.001	**0.830**	<0.001
*A. domesticus*					
Dry mass	Pooled	**0.359**	<0.001	0.147	0.108
	Female	**0.969**	<0.001	**0.951**	<0.001
	Male	**0.437**	0.005	**0.372**	0.004
Fat mass	Pooled	−**0.322**	0.003	−0.124	0.177
	Female	−**0.343**	0.006	−0.185	0.153
	Male	−**0.404**	0.001	−0.189	0.151
Lean dry mass	Pooled	**0.585**	<0.001	**0.414**	<0.001
	Female	**0.955**	<0.001	**0.899**	<0.001
	Male	**0.313**	0.015	0.217	0.098
Water mass	Pooled	**0.806**	<0.001	**0.641**	<0.001
	Female	**0.833**	<0.001	**0.633**	<0.001
	Male	**0.772**	<0.001	**0.636**	<0.001

Our sex-specific analyses in *G. texensis* showed that both condition indices were significantly positively correlated with three of the four scaled mass components in both sexes with 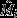 having stronger correlations than *R*_i_. Neither index was significantly correlated with scaled fat mass in females whereas both indices were significantly positively correlated with scaled fat mass in males. Similarly, Gray and Eckhardt (2001) found that *R*_i_ reflected fat reserves in male *G. texensis*, but only when individuals were reared on a poor diet. We also found that in *A. domesticus* 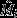 was more strongly correlated with each of the mass components than was *R*_i_ in both sexes but the direction of the relationship varied among the mass components. Only 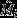 in males was significantly, but negatively, correlated with scaled fat mass.

Our results suggest that 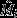, but not *R*_i_, is a suitable index of energetic reserves in *A. domesticu* s with the caveat that it is negatively related to fat content. On the other hand, neither condition index is appropriate in *G. texensis* if the component of interest is energetic reserves (i.e., fat content). Our findings should serve as a warning to biologists that condition indices must be empirically verified rather than assumed.

### Comparison of condition index methods

**Figure 2:**
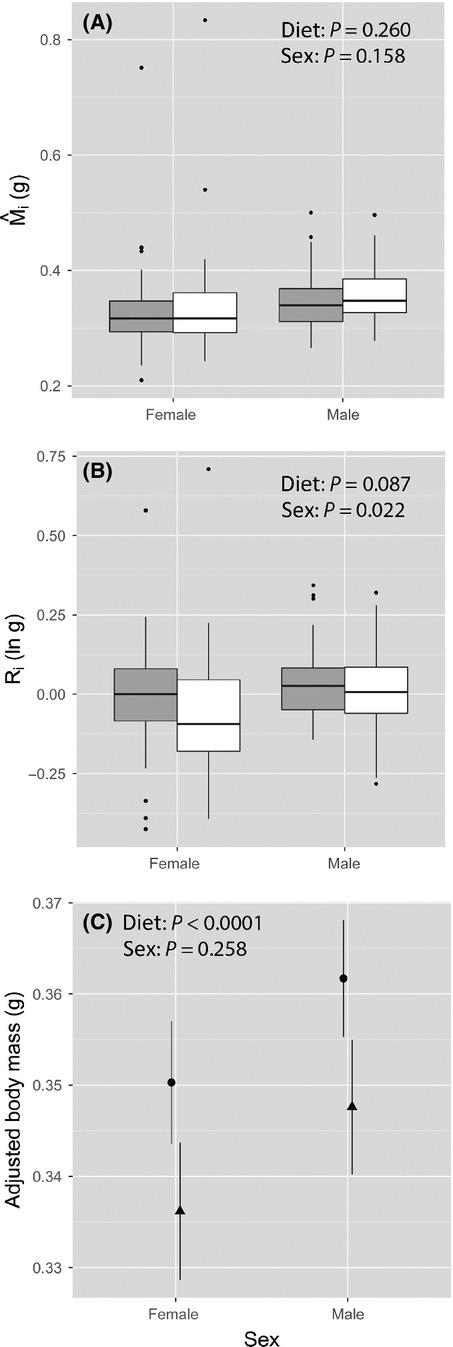
Effect of sex and diet quality (good quality = grey boxes; poor quality = white boxes) on body condition index in *Gryllus texensis* as estimated by three different methods for the Kelly and Tawes (2013) data. Boxplots are shown for the (A) scaled mass index (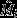) and (B) residual index (*R*_i_). Boxes represent the lower (25%) and upper (75%) quartiles, the solid dark horizontal line represents the median, and the whiskers indicate 1.5 times the interquartile range. Data beyond the end of the whiskers are outliers and plotted as black dots. (C) Mean (±1 SE) body mass (log g) from ANCOVA after adjusting for body size (log mm; circles = good diet; triangles = poor diet). Samples sizes are: low-quality females, *n*  = 35; low-quality males, *n*  = 40; high-quality females, *n*  = 47; high-quality males, *n*  = 52. See Table3 for details of statistical tests.

We used three approaches (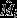, *R*_i_, and ANCOVA) to analyze the effect of two factors (i.e., sex and diet) on body condition in a previously published data set (Kelly and Tawes 2013). These methods produced results that would lead to very different biological interpretations of how sex and diet affect body condition in *G. texensis* crickets at eclosion (Table3; Fig.2). 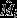
suggests that neither sex nor diet affect body condition, whereas *R*_i_, suggests that males are in significantly better condition than females with diet having a marginally non-significant effect on condition. In contrast, the use of ANCOVA to statistically control for differences in body size among crickets suggests that individuals on a good diet are in significantly better condition than those reared on a poor diet with sex having little effect on body condition.

**Table 3:** Analysis of effect of sex and diet quality on body condition in *Gryllus texensis* using three different condition index methods. Data are from Kelly and Tawes (2013). Bold type indicates a significant factor effect at *α*  = 0.05

Factor	Scaled mass index ( 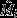 )		Ordinary least squares residual index (*R*_i_)		ANCOVA	
	*F* (df)	*P* -value	*F* (df)	*P* -value	*F* (df)	*P* -value
Sex	2.008 (1,171)	0.158	5.364 (1,171)*	**0.022**	1.29 (1,170)†	0.258
Diet	1.276 (1,171)	0.260	2.959 (1,171)	0.087	132.4 (1,170)†	**<0.001**

* *P*  < 0.05 for the Levene's homoscedasticity test.

† No heterogeneity of slopes in the ANCOVA method at *α*  = 0.05.

We found that SMA better describes the scaling relationship between body mass and body size than OLS regression whether we use the SMA slope from the reference population (2.642 ± 0.11 vs. 2.011 ± 0.088; *z*  = 4.54, *P * < * * 0.001) or from the Kelly and Tawes (2013) data set (2.32 ± 0.088 vs. 2.011 ± 0.088; *z*  = 2.46, *P * = * * 0.014). Using the SMA slope derived from the Kelly and Tawes (2013) data set (i.e., *b*_SMA_ = 2.32) produced results that were very similar (sex: *F * = * * 2.57, df = 1,171, *P * = * * 0.111; diet: *F * = * * 0.001, df = 1,171, *P * = * * 0.980) to those using the slope from the reference population (i.e., *b*_SMA_ = 2.642). This might not always be the case, however, and so we recommend that biologists use *b*_SMA_ from our reference populations in their calculations of 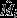 in *G. texensis* or *A. domesticus*.

Prior to calculating *R*_i_ using a common slope (*b*_OLS_) with the Kelly and Tawes (2013) data set, we tested whether the slopes of the groups within each treatment (i.e., males vs. females within “sex” and poor vs. good diets within “diet”) differed significantly (see Garcia-Berthou 2001). The OLS slopes did not differ between the sexes (males vs. females: *b*_OLS_ = 2.16 ± 0.14 vs. *b*_OLS_ = 1.92 ± 0.11, *t * = * * 1.33, df = 170, *P * = * * 0.185) or diets (poor vs. good: *b*_OLS_ = 2.05 ± 0.14 vs. *b*_OLS_ = 182 ± 0.14, *t * = * * 1.16, df = 170, *P * = * * 0.25). We thus pooled the data and calculated *R*_i_ for each individual using a common slope (*b*_OLS_ = 2.01 ± 0.088). Residual body mass was then used to examine the effects of sex and diet on body condition.

**Figure 3:**
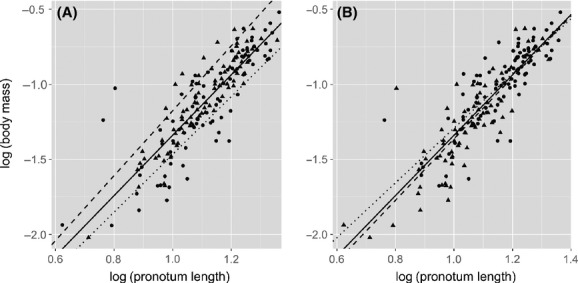
Relationship between body mass (ln g) and body size (ln mm) as described by ordinary least squares (OLS) regression in *Gryllus texensis* for (A) each sex separately (males: triangles and dashed line; females: circles and dotted line) and (B) for each level of diet quality (poor diet: triangles and dashed line; good diet: circles and dotted line) for the Kelly and Tawes (2013) data. The common OLS slope is represented by the solid line in both plots.

Testing the homogeneity of slopes assumption is not the only assumption that requires examination, however, because the elevation of the regression lines also plays an important role in potentially biasing residual calculations and scaling coefficients (Garcia-Berthou 2001). For example, despite there not being an interaction between the sex-specific OLS slopes in the Kelly and Tawes (2013) data set, we found that the male slope had a significantly greater intercept (i.e., elevation) than the female slope (sex: *t * = * * 2.30, df = 171, *P * = * * 0.023). That is, after removing the non-significant interaction and then testing for differences between the sexes in adjusted body mass while statistically controlling for body size (i.e., performing an ANCOVA), we found that the adjusted mean body mass of males was significantly greater than that for females (Fig.3). Thus, our common slope had a lower elevation than the male-specific slope and a higher elevation than the female-specific slope. Ignoring this fact has serious implications for the calculation of residual body mass because the common slope produced residuals that were spuriously large for males and small for females. In contrast, the intercepts (i.e., adjusted means) did not significantly differ between poor and good diets (diet: *t * = * * 1.852, df = 171, *P * = * * 0.066). Consequently, our statistical analysis using *R*_i_ as the condition index suggested that there is a significant difference between the sexes, with diet quality having little effect on condition.

Using *R*_i_ is a well-accepted and established approach in the field of evolutionary ecology despite having several drawbacks (Kotiaho 1999; Garcia-Berthou 2001; Green 2001; Peig and Green 2009, 2010). Our re-analysis of Kelly and Tawes’ (2013) data highlights that OLS regression does not accurately describe the scaling relationship between body mass and body size and that ignoring key statistical assumptions leads to spurious differences between treatment factors.

**Figure 4:**
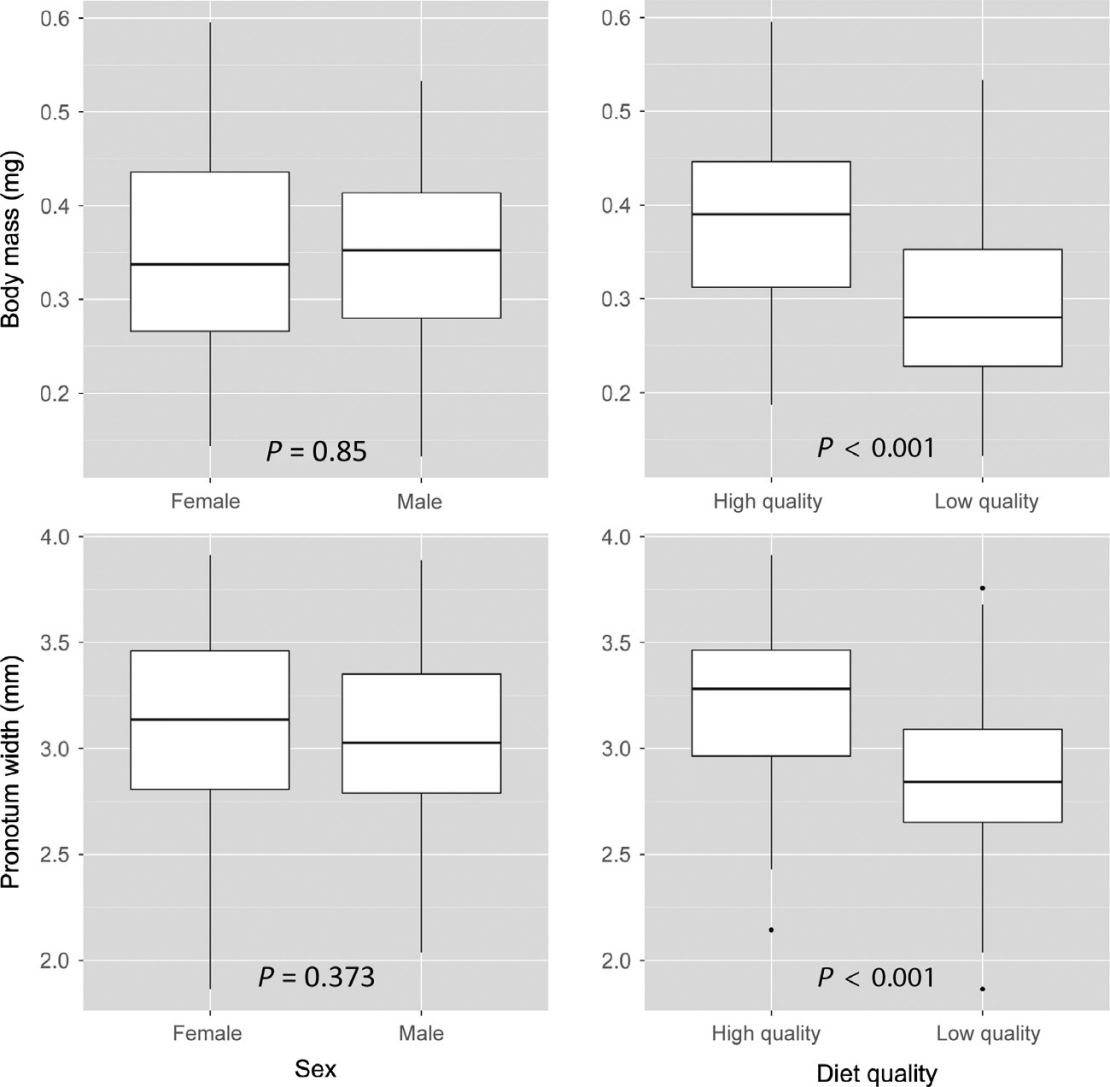
Boxplots showing differences in body mass (g) and body size (pronotum length, mm) between the sexes and levels of diet quality in *Gryllus texensis* for the Kelly and Tawes (2013) data. The box represents the lower (25%) and upper (75%) quartiles, the solid dark horizontal line is the median, and the whiskers indicate 1.5 times the interquartile range. Data beyond the end of the whiskers are outliers and plotted as black dots (females, *n*  = 82; males, *n*  = 92; high-quality diet, *n*  = 99; low-quality diet, *n*  = 75). *P* -values are from one-way ANOVAs testing for differences in mass or size between each treatment level (see text).

Finally, the significant effect of diet using ANCOVA appears to be a result of this method simply tracking the significant differences in body mass and size between the diet treatments (Fig.4). Crickets that were reared on a good quality diet were larger in body mass (*F * = * * 37.07, df = 1,172, *P * < * * 0.0001) and size (*F * = * * 38.28, df = 1,172, *P * < * * 0.0001) than those on a low-quality diet. The sexes did not differ significantly in the size of either trait (body mass: *F * = * * 0.036, df = 1,172, *P * = * * 0.85; body size: *F * = * * 0.80, df = 1,172, *P * = * * 0.37), and consequently, ANCOVA showed no effect of sex on body condition. These findings are similar to those of Peig and Green (2010) who also found that ANCOVA produced significant differences in condition according to differences in body size in several vertebrate taxa.

In conclusion, we showed that the scaling relationship between different body mass components and different measurement of body size varies between species and between the sexes with a species. We strongly recommend that biologists not assume that a particular index is a reliable indicator of body condition but rather they empirically verify the reliability of the condition index. We also show that the best description of the scaling relationship between body mass and body size in both cricket species was produced by SMA, rather than OLS, regression and our values for *b*_SMA_ from our reference populations should be used by biologists when calculating 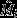 in *G. texensis* and *A. domesticus*. Finally, our re-analysis of the data presented in Kelly and Tawes (2013) illustrates well the dangers of analyzing body condition using methods that are based on least squares in the *y* -plane. Both *R*_i_ and ANCOVA suggested that body condition was significantly affected by a different treatment factor while 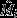 suggested no treatment effects. Thus, depending on the method employed, very different biological conclusions would be drawn from the same data.
